# Robot-Assisted Gait Training in a Patient with Adult Polyglucosan Body Disease: A Case Report

**DOI:** 10.3390/jcm15134996

**Published:** 2026-06-26

**Authors:** Seoyeon Shin, Jeehyun Yoo, Dasom Oh, Jinseong Kim, Jihoon Jeong, Sehaeng Jo, Yeorin Kim

**Affiliations:** 1 Department of Physical Medicine and Rehabilitation, Ilsan Paik Hospital, Inje University, Goyang 10380, Republic of Korea; i8159@paik.ac.kr (S.S.); jhyoo@paik.ac.kr (J.Y.); 2 Academic Cooperation Foundation, Inje University Industry, Gimhae-si 50834, Republic of Korea; dasom654@naver.com; 3 Departments of Physical Therapy, Ilsan Paik Hospital, Inje University, Goyang-si 10380, Republic of Korea; kjincastle@paik.ac.kr (J.K.); i3448@paik.ac.kr (J.J.); i4400@paik.ac.kr (S.J.)

**Keywords:** polyglucosan body disease, adult form, gait, robotics

## Abstract

**Background/Objectives**: Adult Polyglucosan Body Disease (APBD) is a rare neurodegenerative glycogen storage disorder characterized by progressive gait disturbance, sensory impairment, and balance dysfunction. Although rehabilitation is recommended for functional maintenance, evidence regarding robot-assisted gait training (RAGT) in APBD remains extremely limited. **Methods**: A 58-year-old man with progressive lower extremity sensory and motor symptoms was diagnosed with APBD in 2026. Neurological examination revealed severe proprioceptive impairment in both great toes, generalized sensory deficits, gait instability, and impaired balance. Functional assessment demonstrated mild balance impairment with generally preserved muscle strength except for mild weakness in the lower extremities. RAGT was initiated and performed for 19 sessions over approximately 6 weeks in combination with conventional rehabilitation therapy, including gait and balance training with visual feedback. **Results**: Following the combined rehabilitation program, improvements were observed in balance function, postural stability and proprioceptive function. **Conclusions**: This case suggests that RAGT combined with conventional rehabilitation may improve balance and gait-related function in patients with APBD. Repetitive task-specific gait training with enhanced sensory feedback may be particularly beneficial in APBD, where proprioceptive impairment and sensory ataxia are major contributors to gait dysfunction; however, this remains a hypothesis that requires validation in future studies. This report highlights the feasibility and potential applicability of RAGT in rare neurodegenerative disorders such as APBD.

## 1. Introduction

Adult Polyglucosan Body Disease (APBD) is a rare autosomal recessive disorder of glycogen metabolism characterized by the accumulation of poorly branched insoluble glycogen, commonly known as polyglucosan bodies, within the nervous system. This accumulation leads to progressive dysfunction of both the central and peripheral nervous systems, including involvement of the corticospinal tracts, peripheral nerves, and dorsal root ganglia [1]. Deficiency or dysfunction of the glycogen branching enzyme encoded by the *glycogen branching enzyme 1* (*GBE1*) gene causes APBD, leading to the formation of structurally abnormal glycogen that disrupts normal cellular function, particularly in neural tissues [1,2].

APBD is an extremely rare disorder; its precise prevalence in the general population remains uncertain, owing to under-recognition and limited epidemiological data. The overall frequency of glycogen storage diseases is estimated to be approximately 1 in 10,000 individuals, of which GBE deficiency accounts for approximately 3% [3]. Only a relatively small number of cases have been reported worldwide, and the disease is likely underdiagnosed due to its nonspecific and slowly progressive clinical features.

Clinically, APBD typically occurs in adulthood, most often between the fourth and sixth decades of life, and follows a slowly progressive course. The most common initial manifestations include gait disturbance and neurogenic bladder dysfunction. As the disease progresses, patients frequently develop a combination of upper and lower motor neuron signs, peripheral neuropathy, and varying degrees of cognitive impairment. Gait impairment is a predominant feature that often leads to substantial functional limitations and progressively reduced independence.

Neuroimaging findings commonly reveal diffuse white matter abnormalities consistent with leukoencephalopathy, with a predilection for periventricular regions and corticospinal tracts [4,5]. Diagnosis is based on a combination of clinical features, radiological findings, and confirmatory testing, including reduced GBE activity or identifying pathogenic variants in the *GBE1* gene. Polyglucosan body accumulation within the spinal cord, nerve roots, and peripheral nerves leads to mixed upper and lower motor neuron involvement and distal sensory loss, with progressive proprioceptive impairment representing a primary contributor to gait instability in APBD [1,2].

To date, no disease-modifying treatment is available for APBD; its management remains, therefore, largely supportive, focusing on symptomatic relief and preservation of functional abilities. Rehabilitation interventions play a crucial role in maintaining mobility and quality of life. Nevertheless, evidence regarding the effectiveness of advanced rehabilitation strategies, such as robot-assisted gait training (RAGT), in patients with APBD remains limited.

RAGT has been extensively studied for its efficacy in central nervous system disorders such as stroke and spinal cord injury. Various types of RAGT devices are available, including exoskeletal and end-effector systems. End-effector robots, which primarily interact with the patient’s feet, allow relatively greater freedom of movement at the knee and hip joints and enable dynamic training conditions that can challenge postural control. This type of training facilitates neural circuit reinforcement, enhances balance control, and promotes sensorimotor integration [6,7]. The ground-walk mode of these robots simulates level-ground ambulation, and these systems have been shown to produce lower-limb muscle activation patterns comparable to those observed during walking [8].

Although RAGT has been predominantly investigated in patients with central nervous system disorders, its application in rare neurodegenerative diseases, such as APBD, has not been well described. Given that gait dysfunction in patients with APBD is largely associated with impaired proprioception, sensory ataxia, and balance deficits rather than isolated muscle weakness, RAGT may provide therapeutic benefits by offering repetitive task-specific gait training and enhanced external sensory feedback. To our knowledge, no prior case reports have described the application of RAGT in patients with APBD. Therefore, in this case report, we aimed to describe the application of RAGT in a patient with APBD and explore its potential clinical implications.

## 2. Case Presentation

### Methods

A 58-year-old man presented with a history of progressive sensory and motor symptoms in the lower extremities. He first experienced tingling sensations in both lower limbs in 2021. By 2022–2023, he reported rapidly progressive weakness that significantly interfered with occupational activities. In 2023, he was diagnosed with ankylosing spondylitis in the United States and found to be HLA-B27 positive. The diagnosis of ankylosing spondylitis was not revised; however, although HLA-B27 positivity supported this diagnosis, the patient’s progressive sensory deficits, neurogenic bladder dysfunction, and characteristic neurological findings were not adequately explained by ankylosing spondylitis alone and prompted further neurological evaluation. In addition, the patient’s older sister had been diagnosed with APBD and was wheelchair-dependent due to progressive gait impairment, further supporting the likelihood of an underlying hereditary neurodegenerative disorder. In 2026, he was diagnosed with APBD at the Asan Medical Center based on characteristic clinical manifestations, neuroimaging findings, and genetic confirmation of compound heterozygous pathogenic variants in the *GBE1* gene (c.[556-1G>A]; [610G>T], p./splicing; V204L). Brain Magnetic Resonance Imaging (MRI) revealed atrophy of the lower brainstem and cervical spinal cord, bilateral symmetrical T2 hyperintensities in the postero-inferior basal ganglia, and grade II white matter changes. Cervical spine MRI demonstrated diffuse cervical cord thinning with increased signal intensity from the cervicomedullary junction to the C7 level, findings compatible with the central nervous system involvement characteristic of APBD [4,5].

The patient first visited the outpatient clinic of the Department of Rehabilitation Medicine of our institution in February 2026 for functional evaluation and rehabilitation planning. Although lower-extremity spasticity was present, it did not significantly interfere with gait during routine ambulation and tended to worsen only after strenuous physical activity. No definitive upper motor neuron signs, such as hyperreflexia or a Babinski sign, were observed on examination. Therefore, antispastic medication was not used during the rehabilitation period. No other medications known to influence neurological function or rehabilitation outcomes were administered during the intervention period. Formal cognitive assessment was not performed; however, the patient reported subjective memory decline and increased forgetfulness. In addition, the patient had a neurogenic bladder with a large post-void residual volume and required clean intermittent catheterization.

Proprioception of both great toes was assessed using a passive joint movement detection paradigm at the interphalangeal joint, consistent with standard neurological examination technique [9]. With the patient’s eyes closed, the examiner manually moved the great toe upward or downward while grasping the digit from the medial and lateral sides to minimize tactile cues. The patient was asked to identify the direction of each movement. Ten trials were administered per side, consistent with methods described in the proprioceptive assessment literature [10]. The great toe was positioned parallel to the ground and defined the reference position (0°). Using a goniometer, passive movements of 5° and 10° were then applied in both directions. At the initial assessment, the patient was unable to detect passive movements even at displacements of up to 10° in either great toe. However, proprioception was preserved proximally to the ankle. Sensory examination revealed sensory deficits below the L1 dermatome in the lower extremities, with generalized sensory impairment noted in the upper extremities. The patient was able to walk independently, but due to decreased sensation, exhibited poor dynamic balance and walked with an unsteady gait. He also dragged the right leg while walking and frequently reported a sensation of impending falls when walking on uneven surfaces.

A comprehensive functional assessment was performed on 12 March 2026. Motor strength was evaluated using the Medical Research Council (MRC) scale. Upper extremity strength was grade 4 bilaterally, while lower extremity muscle groups ranged from grade 3 to 4. The Berg Balance Scale (BBS) score was 49, indicating mild balance impairment.

RAGT with the Morning Walk^®^ system (Curexo Co., Ltd., Seoul, Republic of Korea), an end-effector-based robotic gait training device, was initiated on 13 March 2026, in ground-walk mode. Unlike treadmill-based exoskeletal robotic systems, the Morning Walk^®^ system generates gait movements through programmable footplates and uses a saddle support mechanism that allows adjustment of body weight support (BWS). Training intensity can be modified by adjusting the degree of BWS, cadence, and gait-training mode.

Because the patient was capable of independent ambulation, training was initiated with 0% BWS and a cadence of 40 steps/min. The patient underwent a total of 19 sessions between 13 March and 23 April 2026, with each session lasting 20 min. Although no consensus guideline exists for RAGT dosing in rare neurodegenerative diseases, the selected regimen of 19 sessions over approximately 6 weeks is comparable to protocols reported in RAGT studies for other neurological conditions [6,7]. The session duration of 20 min was determined based on the patient’s tolerance to intensive gait training, as no standardized session length has been established for RAGT in this patient population. The patient tolerated all 19 sessions without significant adverse events. No musculoskeletal pain, skin breakdown from saddle or footplate contact, or exacerbation of neurological symptoms was observed or reported throughout the intervention period. Mild fatigue was noted following early sessions but did not require session modification or discontinuation. Progression was guided by clinical performance during robotic training. The progression criterion was defined as the ability to complete a continuous 20 min session without rest while maintaining ground reaction force (GRF) above 90% of body weight. At this point, cadence was increased by 5 steps/min, following a stepwise progression approach consistent with previously published Morning Walk^®^ protocols [6]. According to these criteria, cadence was maintained at 40 steps/min during sessions 1–5, increased to 45 steps/min during sessions 6–8, 50 steps/min during sessions 9–10, and 55 steps/min from session 11 onward.

The cadence was maintained at 55 steps/min because a slight decrease in GRF was observed when the cadence was increased from 50 to 55 steps/min, although GRF values remained above 90%. According to our institutional progression criteria, cadence was increased in 5-steps/min increments if GRF values recovered to ≥98% of body weight, a level previously observed at a cadence of 50 steps/min. The long-term target cadence was 70 steps/min.

Conventional rehabilitation therapy was provided immediately after each RAGT session on the same day, for a total of 19 sessions, with each session lasting 20 min. The conventional rehabilitation program included rehabilitative developmental therapy and gait training comprising stepping exercises, balance training with visual feedback, heel raises, and side-stepping exercises. The exercise program was individualized according to the patient’s balance performance and gait stability throughout the intervention period. The duration and frequency of training were determined according to the institutional rehabilitation protocol and the patient’s tolerance to intensive gait training.

**Figure 1:**
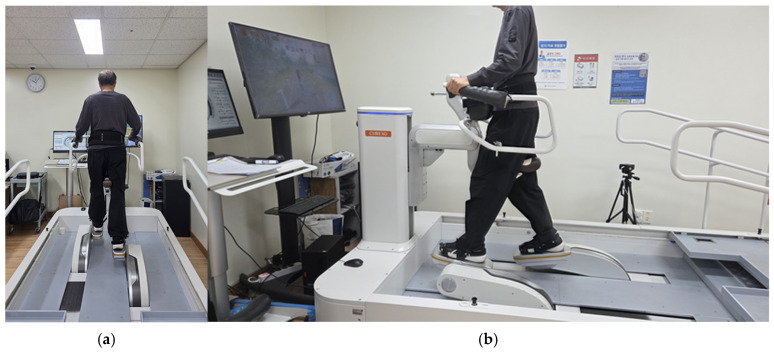
Robot-assisted gait training (RAGT) session. (**a**) Anterior view of the patient performing ground-walk mode training; (**b**) lateral view of the patient during the same training session.

Figure 1 shows the patient performing RAGT sessions from the anterior (a) and lateral (b) views.

## 3. Results

### 3.1. RAGT Gait Parameters

**Figure 2:**
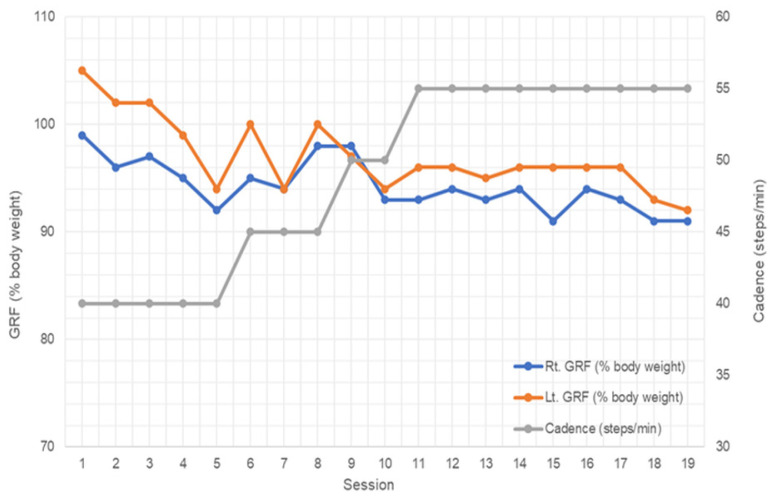
Changes in ground reaction force (GRF) and cadence across 19 robot-assisted gait training (RAGT) sessions in ground-walk mode. Right GRF (blue), left GRF (orange), and cadence (gray) are shown as percentages of body weight and steps per minute, respectively.

Changes in gait parameters during RAGT sessions were monitored throughout the intervention period. GRF and cadence were recorded across all 19 sessions and are presented in Figure 2. GRF data were obtained from the footplate sensors integrated in the Morning Walk^®^ system. The device records three-dimensional GRF (vertical, anterior–posterior, and medio-lateral components) through six-axis load cells; however, only the vertical component is provided in the clinical report. The GRF value used in this study represents the average of the peak vertical GRFs measured during the stance phase across all steps in each training session, expressed as a percentage of body weight. Cadence was automatically recorded by the robotic system during training sessions. No predefined target cadence was established, and cadence values reflected the patient’s performance during robotic gait training. Cadence demonstrated a stepwise increase, stabilizing at approximately 55 steps/min from session 11 onward, suggesting progressive adaptation to the training demands. As cadence increased, the GRF decreased to 90%; however, it did not deteriorate further and remained above 90%.

### 3.2. Functional Outcome Measures

Motor strength was assessed using the MRC scale at baseline (12 March 2026) and follow-up (13 April 2026). At baseline, upper extremity strength was grade 4 bilaterally, while lower extremity muscle groups ranged from grade 3 to 4. At follow-up, motor strength remained largely unchanged, with upper extremity strength maintained at grade 4 bilaterally and lower extremity muscle groups similarly ranging from grade 3 to 4, suggesting overall stable motor function throughout the rehabilitation period.

The BBS was administered at baseline (12 March 2026) and follow-up (11 May 2026). The score improved from 49 at baseline to 56 at follow-up, reaching the maximum score on the scale. This represents a clinically meaningful improvement in functional balance performance following the intervention.

Dynamic posturography was performed at baseline (12 March 2026) and follow-up (7 April 2026) using the Modified Clinical Test of Sensory Interaction on Balance (M-CTSIB) and the Fall Risk Test. The M-CTSIB evaluates sensory integration under varying visual and surface conditions, while the Fall Risk Test provides a composite stability index. Results are presented in Table 1 and Table 2, respectively. Both assessments demonstrated improved postural stability following the intervention, as evidenced by reductions in sway index and improvement in Z scores across multiple conditions. The right leg dragging observed during gait prior to treatment resolved on clinical observation, and the patient also subjectively reported a reduced sensation of feeling likely to fall.

At the outpatient follow-up visit on 28 April 2026, improvement was observed in the patient’s ability to detect passive joint movement of both great toes during follow-up assessment. The patient was able to detect joint position changes at less than 5° of displacement, compared with an inability to perceive movement even at 10° at baseline. This finding suggests a meaningful improvement in proprioceptive function and passive joint position sense following the rehabilitation program.

## 4. Discussion

APBD is a rare, slowly progressive neurogenetic disorder characterized by gait difficulty, distal sensory loss, mixed upper and lower motor neuron involvement, and neurogenic bladder dysfunction. Multidisciplinary symptomatic management is recommended for APBD, including individualized physical therapy aimed at maintaining or improving flexibility, joint mobility, muscle strength, coordination, balance, and activities of daily living [2]. To our knowledge, no previous peer-reviewed case reports have specifically described the application of RAGT in patients with APBD. Literature on the rehabilitation for APBD remains extremely limited. Carneiro et al. [11] reported the case of a 65-year-old woman with APBD who underwent an intensive inpatient rehabilitation program delivered by a multidisciplinary team, emphasizing that functional impairments and rehabilitation approaches for APBD have rarely been reported. Therefore, the present case may provide additional clinical insights into the potential role of structured gait-oriented rehabilitation, particularly RAGT, in patients with APBD.

In this case, a combined rehabilitation program consisting of RAGT and conventional rehabilitation therapy was implemented to manage gait instability, impaired balance, and sensory-related walking difficulties. Although muscle strength remained largely stable throughout the intervention period, the observed improvements in balance performance and postural stability suggest that the benefits of rehabilitation were primarily attributable to enhanced postural control and gait coordination rather than increases in muscle strength. The potential benefit of RAGT in this patient may be related to its ability to provide repetitive, task-specific, and rhythmic gait practice with controlled lower limb movements. Robot-assisted rehabilitation has been widely used in other neurological disorders because it allows intensive and reproducible gait training with feedback, which may help improve walking ability and balance. This mechanism may be particularly relevant in patients with APBD, where progressive sensory loss and proprioceptive impairment could compromise gait stability. In the present case, concomitant visual feedback-based balance training might have helped compensate for impaired proprioception. However, the observed improvement in proprioceptive performance should be interpreted with caution. Given the progressive neurodegenerative nature of APBD and the involvement of large-fiber sensory pathways, true restoration of degenerated proprioceptive afferents is biologically unlikely. Therefore, the observed change should not be interpreted as regeneration of impaired sensory pathways. Rather, it may reflect enhanced utilization of residual proprioceptive input, sensory reweighting, improved central integration of afferent signals, or compensatory strategies facilitated by repetitive gait training and visual feedback. The rehabilitation program incorporated both RAGT and conventional balance training, which may have repeatedly stimulated mechanoreceptors within muscle spindles, Golgi tendon organs, and joint capsules, thereby enhancing proprioceptive input from the foot, ankle, and trunk. In particular, the end-effector-based robotic system provided repetitive, rhythmic, and task-specific gait cycles with consistent ankle and knee movements, potentially promoting repeated activation of residual proprioceptive pathways. Repetitive ankle and knee movements during robotic gait training, together with progressive increases in training intensity and sensorimotor task difficulty, may have facilitated motor learning, sensorimotor adaptation, and more effective utilization of residual sensory input rather than regeneration of impaired sensory pathways [12]. Similar mechanisms have been described in neurological rehabilitation, where functional improvement may occur despite persistent structural deficits through optimization of preserved neural pathways.

In addition to improvements in clinical balance measures, serial gait parameter analysis during RAGT demonstrated progressive increases in cadence and a slight decrease in bilateral GRF values. This finding may reflect improved gait efficiency and postural control rather than reduced weight-bearing capacity. Early during training, excessive vertical loading and exaggerated GRFs may have occurred as a compensatory strategy for impaired proprioception and instability. As gait stability and sensory integration improved, the patient may have adopted a more energy-efficient and coordinated gait pattern with reduced unnecessary loading during the stance phase [13,14]. However, this interpretation remains speculative. Alternative explanations, including adaptation to the robotic device, cadence-related biomechanical changes, and unmeasured variations in training conditions, cannot be excluded.

Several neurophysiological mechanisms may explain the observed improvements despite the progressive nature of APBD. First, sensory reweighting may have played an important role. In patients with impaired proprioceptive input, the central nervous system can increase reliance on alternative sensory information, particularly visual and vestibular inputs, to maintain postural control and gait stability [15]. In the present case, repeated gait practice using RAGT together with visual feedback-based balance training may have facilitated more effective utilization of these preserved sensory modalities.

Second, the repetitive and predictable nature of robotic gait training may have promoted improvements in motor prediction and feed-forward control. Even in the presence of impaired proprioceptive feedback, repeated exposure to consistent gait cycles may help patients develop more accurate internal models of movement, thereby improving gait coordination and stability [16]. Such mechanisms may be particularly relevant in patients with sensory ataxia, in whom motor control increasingly relies on predictive rather than feedback-driven strategies.

Furthermore, APBD involves both central and peripheral nervous system pathology, including corticospinal tract involvement, leukoencephalopathy, peripheral neuropathy, and degeneration of dorsal root ganglia. Therefore, the observed improvements may not be attributable to a single mechanism but rather to adaptive plasticity within preserved components of the sensorimotor system. Intensive task-specific training may strengthen residual neural circuits and optimize the utilization of remaining pathways despite ongoing neurodegeneration [17].

This study has some limitations. Because APBD is a progressive disease and this is a single case report, the observed improvement cannot be attributed solely to RAGT. The patient also received conventional rehabilitation therapy, making it difficult to isolate the specific effects of the robotic intervention. The follow-up period was short; moreover, the long-term maintenance of functional gains remained uncertain. Interpretation of the BBS findings should also be made with caution. Although the patient’s BBS score improved during the intervention period, the baseline score of 49/56 indicated relatively mild balance impairment. Furthermore, the BBS is known to exhibit ceiling effects in individuals with mild to moderate balance deficits, which may limit its sensitivity to detect clinically meaningful changes. In addition, the minimal clinically important difference for BBS has not been established in patients with APBD or progressive sensory neuropathies. Therefore, the clinical significance of the observed change remains uncertain. Future studies should consider incorporating more sensitive measures of gait-related balance, such as the Mini-BESTest or Functional Gait Assessment. Another important limitation is the absence of standardized overground gait outcome measures, such as the Timed Up and Go test, 10-Meter Walk Test, 6-Minute Walk Test, and Functional Gait Assessment. Consequently, the functional significance of the observed changes in robot-derived gait parameters could not be fully verified. Furthermore, as this was a retrospective case report in which assessments were performed as part of routine clinical care rather than according to a predefined research protocol, outcome measures were obtained at inconsistent time points relative to the intervention period (Table 3). These varying assessment intervals introduce uncertainty regarding whether the observed improvements were sustained or fluctuated over time, and this temporal inconsistency should be considered when interpreting the overall functional trajectory.

The proprioceptive assessment also has several methodological limitations. The bedside passive joint movement detection paradigm, while consistent with standard neurological examination practice and the proprioceptive assessment literature, is a relatively crude measure compared to quantitative instrument-based methods such as threshold detection devices or joint position reproduction tests, which would provide greater measurement precision. Examiner blinding was not performed, and inter-rater reliability was not assessed.

Nevertheless, the improvement in the BBS score and postural stability after rehabilitation suggests that RAGT may be a feasible and potentially beneficial therapeutic option for managing gait dysfunction in patients with APBD.

## 5. Conclusions

This case suggests that RAGT combined with conventional rehabilitation may improve balance and postural control in patients with APBD. Given that gait dysfunction in APBD is largely associated with impaired proprioception, sensory ataxia, and balance deficits, repetitive task-specific gait training with enhanced external sensory feedback may provide meaningful therapeutic benefits.

To our knowledge, this is the first reported case describing the use of RAGT in a patient with APBD. Given the extreme rarity of APBD and the absence of previously published reports on robot-assisted rehabilitation in this population, this case contributes novel clinical observations regarding the potential application of RAGT for APBD-related gait dysfunction. It highlights the potential role of advanced rehabilitation strategies in rare neurodegenerative disorders and suggests that RAGT may be a feasible and clinically useful therapeutic option. Further studies should incorporate standardized gait assessments, comprehensive balance measures, and longer follow-up periods to better characterize outcomes and establish evidence-based rehabilitation strategies for APBD. The applicability of RAGT across different stages of disease progression, including patients with more advanced motor impairment requiring BWS, also warrants investigation. Notably, the patient and his older sister carried identical *GBE1* pathogenic variants yet exhibited markedly different functional outcomes. This intrafamilial variability is of particular interest, suggesting that factors beyond genotype, such as modifier genes and comorbidities, may influence disease trajectory in APBD. Given the progressive nature of APBD, further research should also examine multidisciplinary rehabilitation approaches, long-term maintenance of functional gains, and quality of life outcomes in this population.

## Figures and Tables

**Table 1:** M-CTSIB results: Sway Index and impairment (%) at baseline and follow-up.

Condition	Sway Index (Baseline)	Sway Index (F/U)	Impair % (Baseline)	Impair % (F/U)
Eyes Open Firm Surface	1.21	1.16	27%	25%
Eyes Closed Firm Surface	4.07	3.84	66%	63%
Eyes Open Foam Surface	2.83	2.06	49%	30%
Eyes Closed Foam Surface	8.88	8.01	61%	55%
**Composite Score (Avg.)**	**4.25**	**3.77**	**52%**	**45%**

F/U: follow-up; M-CTSIB: Modified Clinical Test of Sensory Interaction on Balance.

**Table 2:** Fall Risk Test results: Sway Velocity Index (SVI) and Z Score at baseline and follow-up.

Condition	SVI (Baseline)	SVI (F/U)	Z Score (Baseline)	Z Score (F/U)
Eyes Open Comfortable Stance	11.36	10.68	2.38	2.05
Eyes Closed Comfortable Stance	19.19	16.39	4.47	3.36
Eyes Open Narrow Stance	19.16	15.45	5.58	3.64
Eyes Closed Narrow Stance	23.60	22.95	5.69	5.42
**Composite Score**	**19.79**	**18.12**	**5.20**	**4.46**

F/U: follow-up; SVI: Sway Velocity Index.

**Table 3:** Timeline of clinical assessments relative to the RAGT intervention period.

Assessment	Date	Timing Relative to Intervention	Notes
Baseline (BBS, MRC, Posturography)	12 March 2026	1 day before RAGT start	Pre-intervention
RAGT intervention period	March 13–23 April 2026	19 sessions, 5×/week	—
Posturography follow-up (M-CTSIB, Fall Risk Test)	7 April 2026	Mid-treatment (~session 7–8)	During intervention
MRC follow-up	13 April 2026	Mid-treatment (~session 10)	During intervention
Proprioception follow-up	28 April 2026	5 days post-treatment	Post-intervention
BBS follow-up	11 May 2026	18 days post-treatment	Post-intervention

BBS: Berg Balance Scale; MRC: Medical Research Council; M-CTSIB: Modified Clinical Test of Sensory Interaction on Balance; RAGT: robot-assisted gait training.

## Data Availability

The data presented in this study are available from the corresponding author upon reasonable request. The data are not publicly available because they contain information that could compromise the privacy of the research participant.

## Abbreviations

The following abbreviations are used in this manuscript:

APBD	Adult Polyglucosan Body Disease
RAGT	Robot-assisted gait training
GBE1	Glycogen Branching Enzyme 1
MRI	Magnetic Resonance Imaging
MRC	Medical Research Council
BBS	Berg Balance Scale
BWS	Body weight support
GRF	Ground reaction force
M-CTSIB	Modified Clinical Test of Sensory Interaction on Balance
SVI	Sway Velocity Index
